# Tagged MRI Based Cardiac Motion Modeling and Toxicity Evaluation in Breast Cancer Radiotherapy

**DOI:** 10.3389/fonc.2015.00009

**Published:** 2015-02-03

**Authors:** Ting Chen, Meral Reyhan, Ning Yue, Dimitris N. Metaxas, Bruce G. Haffty, Sharad Goyal

**Affiliations:** ^1^Department of Radiation Oncology, Rutgers Cancer Institute of New Jersey, New Brunswick, NJ, USA; ^2^Department of Computer Science, Rutgers University, Piscataway, NJ, USA

**Keywords:** tagged MRI, radiation toxicity, cardiac modelling, breast cancer, radiation therapy

## Introduction

Recent research showed radiation for breast cancer can increase heart risks (1, 2). In Ref. (2), it has been noted that for every Gy of radiation a women’s heart risk rises 7.4%. However, the correlation between radiation dose and heart tissue damage is still an open problem. A more accurate model of heart damage will significantly improve the heart safety for patients underwent radiotherapy.

Modern radiation treatment planning systems (TPS) use computed tomography (CT) images for dose calculation and evaluation. For evaluation of heart toxicity from radiotherapy, the dose-volume histogram (DVH), which is generated by overlying radiation dose distribution on heart delineations in CT images, is widely used. However, there are three major factors that deteriorate the accuracy of TPS-calculated heart dose distribution. First conventional CT is a fundamentally static imaging modality without the capability to capture and depict the cardiac motion. Instead, heart is usually blurred in CT images due to the motion artifacts. Second, without special contrast dye, CT provides limited contrast between blood in heart chambers and the surrounding myocardium. The heart region in TPS is actually a mixture of myocardium and blood, although only the radiation dose to the myocardium is accountable for heart risks. Finally, there is significant intra- and inter-fractional heart motion. As heart beats involuntarily during and between radiation treatments, myocardium deforms and moves non-rigidly against the fixed radiation beam so that the static dose distribution calculated in CT based TPS does not reflect the accurate radiation dose distribution in heart.

There is also concern on the choice of the heart function for the evaluation of radiation damage. Based on radiation beam geometry, only part of the heart will receive clinically significant level of radiation during breast cancer treatment. It is possible that the global heart function remains stable temporarily while cells in the irradiated part of the myocardium lose part or all of their functions. In this case, regional heart function, which can be derived from regional heart wall motion and strain analysis, is a better indication of heart damage corresponding to radiation dose.

Although cardiac MRI is widely used in radiology for the diagnosis of heart disease, its application in radiation treatment planning is limited. For multiple reasons, it is not practical to use MRI directly for radiation treatment planning of breast cancer patients. However, via multimodality deformable image registration (DIR) between MRI and CT, MRI images may play a more critical role in the evaluation of the heart damage from whole breast radiation.

Tagged MRI (tMRI) (3) is a relatively new imaging protocol that has been implemented in the detection and diagnosis of regional heart functional loss. tMRI methods record regional heart wall motion information as they create identifiable landmark bands (tags) in the myocardium to establish dense point to point correspondence between images. ECG-gated tMRI image sets can be acquired at different phases of the cardiac cycle using the corresponding pulse sequence. The 4D (3D plus time) cardiac motion model can be retrieved by image registration between tMRIs at different phases.

In the following sessions, we use tMRI as an example to explain how additional heart function information in MRI is retrieved. It is our objective to demonstrate the additional information retrieved from MRI can help the evaluation and protection of heart risks for breast cancer patients, and we want to discuss the possibility of using MRI to establish a more accurate correlation between regional heart functional loss and radiation dose.

## Method

### Heart motion uncertainty in CT

First, we analyze the uncertainty in the CT based TPS-calculated radiation dose distribution of heart. The cardiac motion artifacts in CT acquisition has been previously studied (4) so we focus on the uncertainty related to the intra- and inter-fractional cardiac motion and location variation.

We used kV fluoroscopy imaging to monitor the intra-fractional cardiac motion during breast cancer treatment (experiment A). For a group of 10 left breast cancer patients without breath holding or external breath suppression, fluoroscopy was acquired weekly at the gantry angle of the treating beam for 15 s at 8 fps. The fluoroscopy radiation dose to the patient was clinical insignificant.

To estimate the inter-fractional heart location variation, we registered the weekly CBCT of two t-spine patients (experiment B). CBCT images were registered to match the left breast and the variation of the heart location was evaluated by measuring the average distance of the heart surface in the registered image.

### Cardiac motion retrieval from MRI

For preliminary research purpose, we retrospectively studied two sets of anonymous tMRI data acquired using the Spatial Modulated Magnetization (SPAMM) pulse sequence. Both tMRI sets were ECG-gated and acquired at 24 phases during the cardiac cycle. Each tMRI sets included three long axis (LA) image sets (corresponding to the two chamber, three chamber, and four chamber view), a short axis (SA) image set, and an ECG-gated non-tMRI image set acquired at the end of diastole. The slice thickness of SA tMRI was 5 mm. The spacing between tags was 8 mm. There were both horizontal and vertical tags in the image.

**Figure 1:**
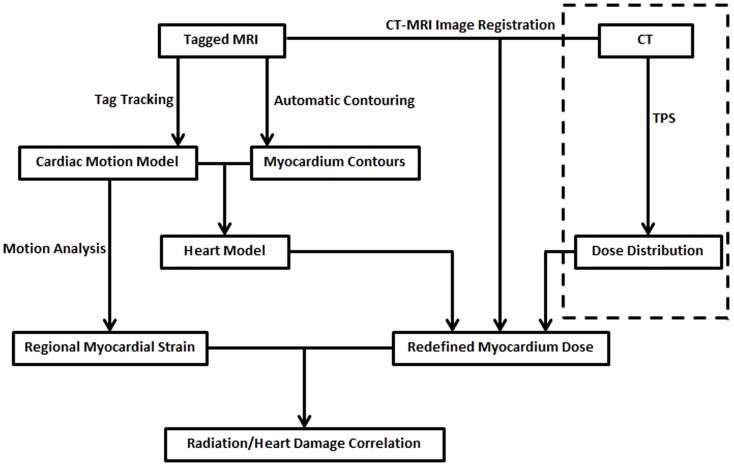
**The work flow of the cardiac motion tracking and myocardium dose evaluation method**. The part encircled by the dashed line is the current CT based heart dose calculation and evaluation in the treatment planning system.

Given tMRI and the corresponding CT images of the breast cancer patient, the work flow to estimate the correlation between radiation dose and regional heart functional loss is illustrated in Figure 1.

The tMRI images went through preprocessing first to remove the intensity non-uniformity introduced by the surface coils used in the MRI process, and to reduce the impact of the decay of image intensity between different phases of the cardiac cycle.

The epi- and endo-myocardial contours were generated from tMRI using frequency domain analysis. The modulated tags corresponded to high frequency components in the frequency domain and can be effectively removed from the image using frequency filtering. We segmented the myocardium automatically in the SA image using the method in Ref. (5). The myocardium contours could be automatically or manually generated in the LA images.

There were multiple means to track the movement of the tags during the cardiac cycle, such as active contours (6), B-Spline (7), physics deformable models (8), and meshless deformable model (9).

The reconstructed cardiac motion model had two uses. First, the myocardial strain distribution was derived from the myocardium motion, and the abnormality in the distribution was used as the indication of local heart tissue damage. Second, the cardiac model was integrated with the CT-MRI image registration to calculate the accumulative radiation dose distribution in the myocardium during cardiac cycle. The radiation dose to the blood was ignored, as it would not directly cause heart risks.

Given the motion distribution, the myocardial strain was computed as the derivative of the displacement vectors at image pixels. Strain depicted the variation in motion between different parts of the heart. Abnormalities (either high or low value) in strain distribution reflected local myocardial motion abnormalities, which was a direct indication of regional heart functional loss.

The CT-MRI image registration was conducted to build a connection between the TPS and the tMRI image domain. First, we register CT to the end-of-diastole non-tMRI using mutual information based multimodality image fusion. The 4D heart model (including both the cardiac motion and the volumetric myocardium model) in the MRI with regard to the beam geometry in the CT images was determined after the registration. In the next step, we accumulated the myocardial dose distribution at different cardiac phases to reconstruct the cardiac-motion-adjusted accumulative myocardium dose distribution. The final step was to align the strain distribution to the motion adjusted dose distribution. The correlation between the strain and radiation dose was calculated to enable us to establish the radiation-dose-to-heart-risk model in future research.

## Discussion

The uncertainty in the TPS dose distribution caused by CT imaging was not considered explicitly in previous studies. The cardiac motion artifacts in CT imaging can be reduced after using modern technology such as the multi-detector computed tomography (MDCT), however currently the high cost limited wide use of such techniques at radiation oncology clinics. The low blood-to-tissue contrast in CT can be increased by injecting contrast dye during patient simulation, although this requires longer preparation time and the improvement is limited if the imaging motion artifact was not well addressed. Based on experiment A, the average infra-fractional motion of the heart wall, as projected to the beam eye view in kV fluoroscopy, was 1.3 ± 0.3 cm. Average inter-fractional heart location variation can be as much as 1.5 cm as measured in the CBCT images acquired in experiment B.

To address the uncertainties in heart location and the corresponding dose distribution, we proposed to use MRI in the evaluation of heart risks for breast cancer patients. As demonstrated by the tMRI-based cardiac analysis framework, MRI had less motion artifacts, higher blood-to-tissue contrast (by using appropriate pulse sequence), and provided infra-fractional cardiac motion information.

The accuracy of the MRI-based cardiac analysis was determined by the accuracy of fundamental image processing modules such as registration, segmentation, and motion tracking. Multimodality image registration between CT and MRI was a well-studied problem and commercial software is now available to generate satisfactory registration results. However, it should be noted that the couch top used in radiation oncology CT simulator, and diagnosis MRI, were different. A DIR should be conducted to correct for the variation of anatomy caused by different couch tops. It was also critical that the registration should align the surface markers in the CT and MRI images since they determined the radiation beam geometry in breast cancer radiation treatment. Effective approaches to automatically delineate the myocardium and to derive the strain from MRI images have been proposed in previous studies. The image registration and motion tracking uncertainties have been discussed in previous research efforts (9, 10). The overall uncertainty in the proposed methodology also depended on the interpolation and extrapolation error during the projection process to transfer the displacement and the radiation dose distribution between different image domains. Interpolation and extrapolation errors were hard to quantify or validate directly. We can use the inverse minimization procedure to reduce the error, at the cost of extra processing time.

The major technical challenge in MRI-based heart risk analysis was the reconstruction of the cardiac-motion-adjusted accumulative radiation dose distribution. To get the accumulative dose, one needed to deform the original CT image to regenerate CT images at different cardiac phases using the cardiac motion derived from the tMRI images. The difficulty increased as the CT and the tMRI imaging planes were not the same and intersected each other at oblique angles. The accuracy of the regenerated CT images needed further validation before using for dose recalculation.

Given adequate information, a polynomial fit can be generated to describe the correlation between regional heart function loss and the radiation dose. The fitted model can be used to quantitatively estimate the heart risk based on accumulative radiation dose.

Finally, it should be noted that although the motion adjusted radiation dose distribution is more accurate and specific than the currently used heart DVH in treatment planning CT, it was still an approximation to the actual dose distribution. The method we proposed did not consider inter-fractional heart location variation and the impact of respiration on heart location. Moreover, the patient heart beat pattern may change during the course of radiation treatment. All these factors caused extra uncertainties in the calculated accumulative myocardium dose.

## Conclusion

We discussed the uncertainties of using CT calculated dose to evaluate the radiation damage to the heart. To improve the quality of heart risk analysis for breast cancer patients, we proposed a tMRI-based framework to derive the cardiac motion, the myocardium strain, and eventually the regional heart function loss. The proposed framework demonstrated the possibility and technical challenge of establishing a correlation between myocardium damage and radiation dose for breast cancer patients using MRI. By using MRI, regional heart function loss could be detected and the radiation dose can be adjusted by generating the accumulative dose during cardiac cycle. We plan to collect tMRI data from more patients to improve the accuracy, efficiency, and statistical robustness of the proposed framework in future studies.

## Conflict of Interest Statement

The authors declare that the research was conducted in the absence of any commercial or financial relationships that could be construed as a potential conflict of interest.
